# Comparative Efficacy of Acupuncture Therapy in Primary Essential Tremor: A Network Meta-Analysis and Systematic Review

**DOI:** 10.3390/healthcare14060803

**Published:** 2026-03-21

**Authors:** Qingping Shi, Jieru Han, Beiyan Chen, Shuang Gao, Mingli Shen

**Affiliations:** School of Basic Medical Sciences, Heilongjiang University of Chinese Medicine, Harbin 150040, China; sqp17326705862@163.com (Q.S.); chenbeiyanabc@163.com (B.C.); gaoshuangacc@163.com (S.G.); 18309474893@163.com (M.S.)

**Keywords:** acupuncture, moxibustion, scalp acupuncture, essential tremor, efficacy rate

## Abstract

Background: Essential tremor (ET) is a common movement disorder that predominantly affects older adults, with rising global prevalence due to population aging. Pharmacological treatments, including propranolol and primidone, are often limited by inadequate efficacy or poor tolerability, and surgical options carry inherent risks. Acupuncture has shown promise as an alternative or adjunctive therapy for ET, but evidence comparing the effectiveness of different acupuncture modalities remains limited. Objective: To systematically evaluate the comparative efficacy and safety of various acupuncture-related interventions for essential tremor (ET) through a network meta-analysis, and to provide evidence-based recommendations for clinical practice. Methods: We systematically searched eight electronic databases (PubMed, EMBASE, Web of Science, Cochrane Library, CNKI, VIP, Wanfang, and CBM) from inception to 20 October 2025. Randomized controlled trials (RCTs) evaluating any form of acupuncture therapy for ET were included. Conventional pairwise meta-analysis and network meta-analysis were performed to compare the efficacy (response rate, Tremor Six Score) and safety (adverse events) of different interventions. Surface under the cumulative ranking curve (SUCRA) values were used to rank treatment modalities. Results: Twenty randomized controlled trials involving 1067 participants were included. Traditional meta-analysis indicated that acupuncture-related interventions significantly outperformed controls in improving response rate [RR 4.36, 95% CI (3.14, 6.03), *p* < 0.00001], reducing Tremor Six Score [MD −1.99, 95% CI (−2.25, −1.73), *p* < 0.00001], and lowering the incidence of adverse events [RR 0.13, 95% CI (0.07, 0.25), *p* < 0.00001]. Network meta-analysis based on SUCRA values revealed that: for symptom relief, scalp acupuncture (S) demonstrated the highest effectiveness (SUCRA = 81.5%); for reducing Tremor Six Score, manual acupuncture (A) showed the most significant effect (SUCRA = 76.6%); and for safety outcomes, Acupuncture + Scalp Acupuncture + Propranolol (A+S+P) achieved the highest SUCRA score (SUCRA = 73.1%). Conclusions: This network meta-analysis demonstrates that acupuncture-related interventions are effective and safe for treating essential tremor. However, caution is warranted in interpreting these findings due to methodological limitations in the included randomized controlled trials (small sample sizes, lack of blinding, inadequate allocation concealment), sparse data for some interventions, and the concentration of studies within China, which limits their generalizability. Despite these limitations, acupuncture offers a valuable non-pharmacological treatment option for patients with poor medication tolerance. Future large-scale, multicenter trials with rigorous designs are needed to validate these findings.

## 1. Introduction

Essential tremor (ET), also known as idiopathic tremor, is one of the most common movement disorders, characterized clinically by bilateral upper limb action tremor, which may be accompanied by tremor in the lower limbs, head, face, or voice [1,2]. The prevalence of ET increases markedly with age, affecting approximately 5.79% of individuals aged 65 years and older, with a 74% increase in prevalence per decade of life. Gender does not influence disease incidence [3]. As the global population ages, the societal burden associated with ET continues to grow substantially.

The pathophysiological mechanisms of ET remain incompletely understood, with three primary hypotheses currently proposed: (a) the neurodegenerative hypothesis, (b) the central oscillatory network hypothesis, and (c) the GABAergic hypothesis [4,5]. Deficiency of the neurotransmitter gamma-aminobutyric acid (GABA) is considered closely associated with essential tremor [6], while enhanced activity in the cerebellar-thalamic-cortical circuit has been demonstrated to be related to the mechanism of tremor onset [5]. Neuroimaging and neuropathological studies reveal structural and functional alterations in the cerebellum, brainstem, and thalamus, supporting the theory that essential tremor primarily involves cerebellar pathology [7].

Standard pharmacological treatment for essential tremor primarily involves non-selective beta blockers such as propranolol and primidone, both considered first-line therapies. Propranolol is the only drug approved by the U.S. Food and Drug Administration (FDA) for treating essential tremor [8]. However, 30–50% of patients fail to respond adequately to these medications, and long-term adherence is poor—approximately 64% discontinue pharmacotherapy within two years due to inadequate efficacy or intolerable side effects [9,10]. Common adverse reactions to propranolol include bradycardia and bronchospasm [11]. For medication-refractory cases, surgical interventions such as deep brain stimulation (DBS), thalamotomy, and MRI-guided focused ultrasound (MRIgFUS) may be considered [8,9]. Despite significant efficacy, these invasive procedures are associated with risks such as intracranial hemorrhage, infection, and speech or gait disturbances, and are not suitable for all patients [12].

Acupuncture, as an integral component of traditional Chinese medicine, is increasingly applied in the treatment of movement disorders including essential tremor. The mechanism of acupuncture therapy for ET may involve regulating gamma-aminobutyric acid (GABA) activity and modulating the cerebellar-thalamic-cortical circuit [13,14,15], thereby acting upon the aforementioned pathophysiological pathways.

Although a previous meta-analysis demonstrated that acupuncture combined with medication was more effective than medication alone for treating essential tremor [16], significant variations in clinical practice exist due to the diversity of acupuncture techniques, and direct comparative evidence between different acupuncture methods remains scarce. Consequently, clinicians lack clear guidance when selecting the most effective acupuncture regimen for individual patients.

To address this evidence gap, we conducted a network meta-analysis (NMA) to systematically compare and evaluate the efficacy and safety of various acupuncture interventions for essential tremor. By integrating direct and indirect evidence, this study aims to provide comprehensive evidence-based guidance for clinical decision-making and optimize treatment strategies for patients with essential tremor.

## 2. Methods

Meta-analysis Registration

Guided by the Cochrane Handbook for Systematic Reviews and Interventions (6th Edition) [17] and the PRISMA guidelines [18], this meta-analysis was conducted. The study protocol was prospectively registered on PROSPERO (CRD420251248029). https://www.crd.york.ac.uk/PROSPERO/view/CRD420251248029, accessed on 6 December 2025.

### 2.1. Inclusion and Exclusion Criteria

#### Article Inclusion Criteria

Only randomized controlled trials (RCTs) assessing different forms of acupuncture therapy for essential tremor (ET) were included. Studies such as conference papers, case reports, animal research, and quasi-randomized trials were excluded due to their non-randomized designs. Cases were included regardless of their source, gender, age, ethnicity, or onset time.

### 2.2. Intervention Types

We included commonly used acupuncture therapies such as manual acupuncture (MA), electroacupuncture (EA), auricular acupuncture (AA), or acupuncture with subcutaneous thread implantation (ACE), either used alone or in combination. Additionally, we considered treatments combining acupuncture with conventional drug therapy, acupuncture with Chinese herbal medicine, and acupuncture with Tuina massage and Chinese herbal medicine.

### 2.3. Intervention Inclusion and Exclusion Criteria

Studies were eligible for inclusion if they met the following definitions for interventions:

1. Intervention group: Any form of acupuncture therapy as defined in Section 2.2, or a combination of acupuncture with other Traditional Chinese Medicine therapies (such as herbal medicine or tuina).

2. Control group: Could include, but was not limited to, placebo/sham acupuncture, no treatment (blank control), conventional Western medication, or other forms of acupuncture therapy that differed in type from the intervention administered in the experimental group.

Studies were excluded if they met any of the following criteria:

1. Lack of a parallel control group: The study design was a non-controlled case series or case report.

2. Ineligible intervention in the experimental group: The experimental group did not include any form of acupuncture therapy as defined in Section 2.2.

3. Ineligible control group: The control group received conventional Western medication without the experimental group concurrently receiving acupuncture therapy (i.e., studies comparing Western medication alone vs. Western medication alone were excluded).

4. Insufficient description of the intervention: The study failed to provide an adequate description of the acupuncture intervention to determine whether it met the review’s definition of acupuncture.

5. Duplicate publication or inability to extract data: Studies that were duplicate publications or from which relevant outcome data could not be extracted.

### 2.4. Search Methods

We searched PubMed, EMBASE, Web of Science, Cochrane Library, China National Knowledge Infrastructure (CNKI), VIP, Wanfang, and China Biomedical Database (CBM). To ensure comprehensive coverage, studies published from the inception of each database up to 20 October 2025, were included without geographical or language restrictions. The search strategy incorporated a combination of terms related to the condition and the interventions. For essential tremor (ET), these included “Essential Tremor”, “Essential Tremors”, and “Benign Essential Tremor”. For acupuncture-related therapies, the terms encompassed “acupuncture”, “moxibustion”, “electroacupuncture”, “warm needle therapy”, “plum-blossom needle”, “three-edged needle”, “fire needle”, “acupoint injection”, and “acupoint catgut embedding”. The search was restricted to randomized controlled trials. The complete search strategy for each database is detailed in Supplementary Materials File S1.

### 2.5. Data Extraction

**Figure 1:**
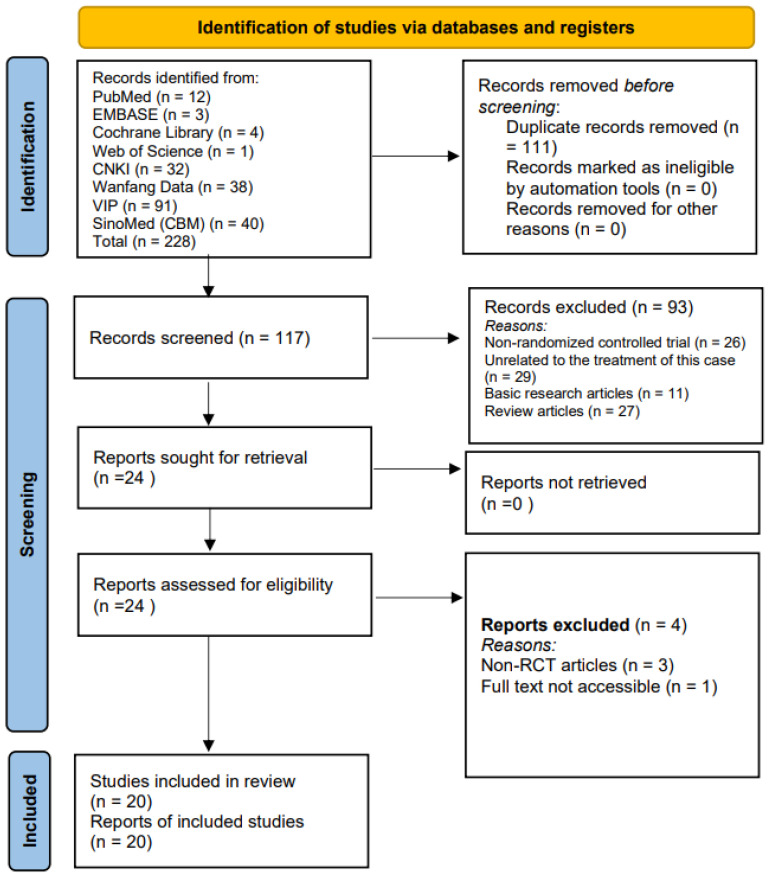
Flowchart of the Research Selection Process.

The study selection was conducted by two reviewers (Qingping Shi and Beiyan Chen). They initially screened studies independently based on titles and abstracts. Then, the full records of studies deemed eligible at this stage were imported into NoteExpress, and duplicates were removed through a collaborative checking process. The full texts of the studies identified as potentially eligible were then independently evaluated by the two reviewers (Qingping Shi and Beiyan Chen) for compliance with the inclusion and exclusion criteria. A flowchart of this selection process is provided in Figure 1. Discrepancies in their assessments were first addressed via discussion between them. If no agreement was reached, the issue was adjudicated by a third reviewer (Jueru Han).

### 2.6. Data Extraction and Analysis

Two reviewers (Qingping Shi, Beiyan Chen) extracted information, including table data such as: first author name, publication year, sample size, gender distribution, mean age, treatment duration and frequency, details of intervention and control groups, specific outcomes included, and any reported adverse events. All collected information was recorded in an Excel spreadsheet.

### 2.7. Methodological Quality Assessment of Included Studies

Two reviewers (Qingping Shi and Beiyan Chen) independently assessed the risk of bias for each included study using the Cochrane Risk of Bias tool. The Cochrane risk-of-bias tool [19] was applied for evaluation. This covered seven key domains: random sequence generation, allocation concealment, blinding of participants and personnel, blinding of outcome assessment, incomplete outcome data, selective reporting, and other biases. Disagreements between reviewers were resolved through discussion. Any disagreements between assessors (Qingping Shi, Beiyan Chen) were determined by communication with the corresponding author (Jieru Han).

### 2.8. Data Analysis

All meta-analyses were conducted employing Stata (version 18.0) and RevMan (version 5.4) software. The inverse-variance method was employed to pool direct and indirect evidence via a frequentist model, weighting studies by their precision (inverse variance) and thereby calculating the weighted mean difference across the network. Based on the inverse of the total weight, the overall effect variance was calculated. After completing the initial meta-analyses, network graphs were then built to facilitate direct comparisons across intervention strategies. In terms of data synthesis, binary outcomes were evaluated using odds ratios (OR), and continuous outcomes were analyzed employing weighted mean differences (MD). Treatment effects are presented as point estimates accompanied by 95% confidence intervals. The 95% confidence interval represents the margin of error for our effect estimates. As this is a meta-analysis of existing studies, no a priori power analysis was conducted. Furthermore, to rank the competing interventions, we applied the Surface Under the Cumulative Ranking (SUCRA) method, which provides a probabilistic hierarchy, and generated the corresponding cumulative probability plots. The SUCRA value, representing the area under the cumulative ranking curve (ranging from 0% to 100%), provides a probabilistic ranking where a higher SUCRA value indicates a greater likelihood of being the most effective treatment [20]. All tables presented in this paper, including the study characteristics table and the summary of findings table, were compiled using Microsoft Excel (version 16.0), and the final output of the pooled effect estimates was formatted using RevMan (version 5.4). The league table showing all pairwise comparisons of interventions was generated using Stata (version 18.0).

Regarding graphical presentations, all forest plots and funnel plots for pairwise meta-analyses were generated using RevMan (version 5.4). The network plot illustrating the geometry of treatment comparisons was constructed using Stata (version 18.0). The comparison-adjusted funnel plots for assessing publication bias in the network meta-analysis were generated using Stata (version 18.0). Additionally, the cumulative ranking probability plots (SUCRAs) were generated using Stata (version 18.0) after fitting the network meta-analysis model.

### 2.9. Publication Bias

We generated comparison-adjusted funnel plots using STATA 18 software to assess publication bias, where asymmetry may indicate publication bias or small-study effects [21]. No studies were excluded based on this assessment.

### 2.10. Sensitivity Analysis

Where significant heterogeneity was present, sensitivity analyses were conducted using STATA 18.0. These analyses aimed to both identify outlier studies that might be driving heterogeneity and explore potential sources of heterogeneity based on study characteristics.

## 3. Results

### 3.1. Search Results

A total of 228 studies were retrieved from eight electronic databases. After removing duplicates, 117 records remained. Screening titles and abstracts led to the exclusion of 93 records (26 non-RCT articles, 29 articles not applicable to treating this disease, 11 basic research studies, and 27 review articles). Full-text review removed 4 records (3 non-RCTs, 1 unavailable for full text). Ultimately, 20 studies were included (details in Figure 1).

### 3.2. Study Characteristics

Included 20 randomized controlled trials (RCTs) involving a total of 1067 patients, published between 2006 and 2024. The sample sizes of the trials ranged from 26 to 80 participants, and the duration of treatment ranged from 15 days to 3 months.

Adverse events: Five studies [22,23,24,25,26] reported the incidence of adverse reactions.

Methodological quality: Seven studies [27,28,29,30,31,32,33] did not clearly specify the method used to generate the random sequence. None reported on allocation concealment. One study [23] reported on loss to follow-up.

**Figure 2:**
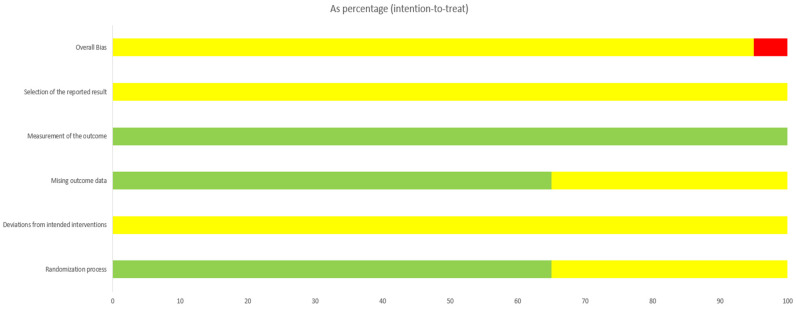
Bias Risk Assessment.

**Figure 3:**
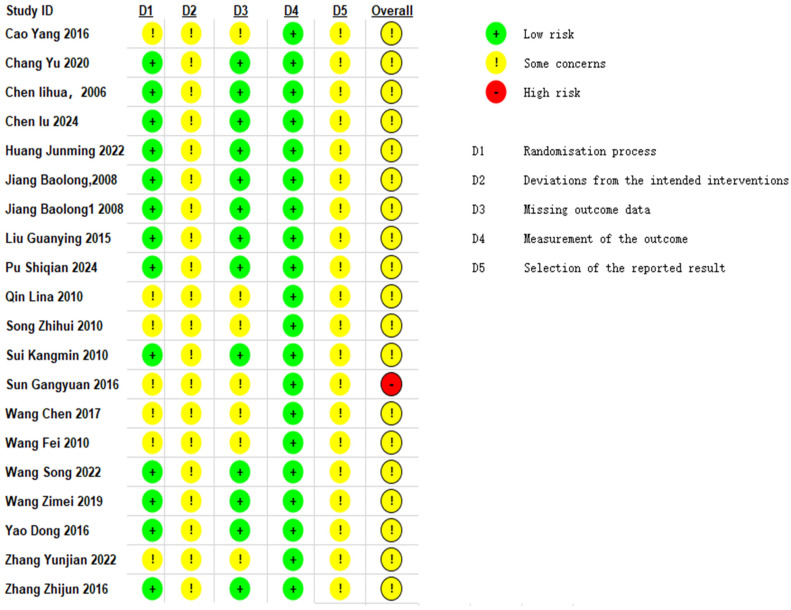
Bias Risk Assessment. D1: Bias arising from the randomization process. D2: Bias due to deviations from intended interventions. D3: Bias due to missing outcome data. D4: Bias in measurement of the outcome. D5: Bias in selection of the reported result. Green indicates low risk. Yellow indicates some concerns. Red indicates high risk [22,23,24,25,26,27,28,29,30,31,32,33,34,35,36,37,38,39,40,41].

Risk of Bias: Figure 2 and Figure 3 present the results of the risk of bias assessment.

Baseline Characteristics: Baseline characteristics were consistent across all patients, with no significant differences observed prior to intervention (see Table 1).

### 3.3. Network Diagram of Different Interventions

**Figure 4:**
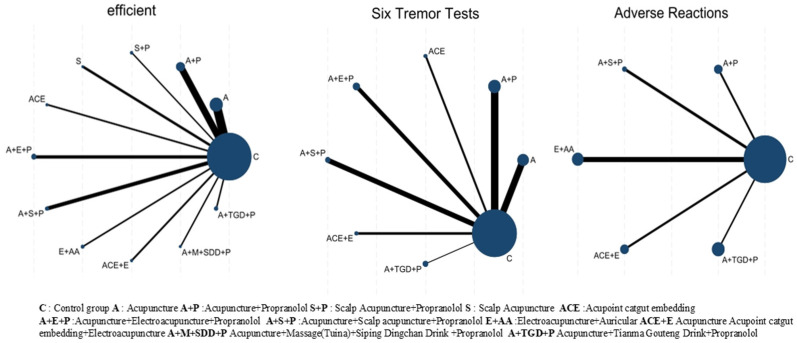
Network Diagram of Different Intervention Measures.

All 20 studies used a two-arm design. Figure 4 presents a network plot showing comparisons between interventions. In this plot, node size reflects the number of participants per treatment, and line thickness indicates the amount of direct evidence for each comparison.

### 3.4. Efficacy Results

**Figure 5:**
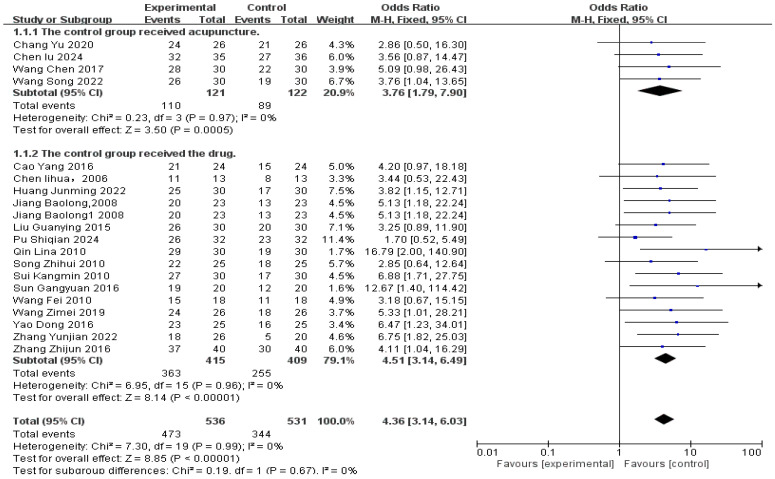
Efficacy-related diagram [22,23,24,25,26,27,28,29,30,31,32,33,34,35,36,37,38,39,40,41].

Response rate was analyzed across all 20 studies, including a total of 536 patients in the acupuncture-related treatment groups and 531 patients in the control groups (Figure 5).

Overall, acupuncture-related interventions showed significantly higher efficacy than control groups, with a response rate approximately four times higher [RR 4.36, 95% CI (3.14, 6.03), *p* < 0.00001]. No significant heterogeneity was detected (*p* = 0.99, I^2^ = 0%), allowing use of a fixed-effects model.

Per-treatment analysis (Table 2) identified nine effective modalities, with the largest effects observed for Scalp Acupuncture (S) [OR = 12.67, 95% CI (1.40, 114.42)], Acupuncture + Massage + Siping Dingchan Drink + Propranolol (A+M+SDD+P) [OR = 6.75, 95% CI (1.82, 25.03)], and Acupoint Catgut Embedding + Electroacupuncture (ACE+E) [OR = 5.33, 95% CI (1.01, 28.21)]. All other significant odds ratios ranged from 3.40 to 5.25.

Two modalities showed no statistically significant difference compared to control: Acupoint Catgut Embedding (ACE) [OR = 2.85, 95% CI (0.64, 12.64)] and Electroacupuncture + Auricular Acupuncture (E+AA) [OR = 3.56, 95% CI (0.87, 14.47)].

**Figure 6:**
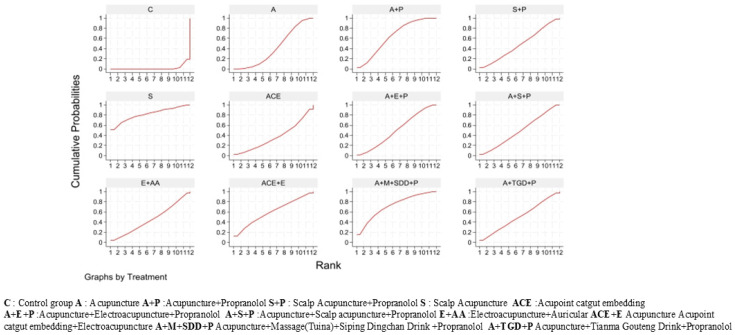
Efficacy SUCRA diagram.

Based on SUCRA values, all acupuncture-related interventions ranked higher than control (C, 2.0%). Scalp Acupuncture (S, 81.5%) was the top-ranked intervention (Figure 6).

**Figure 7:**
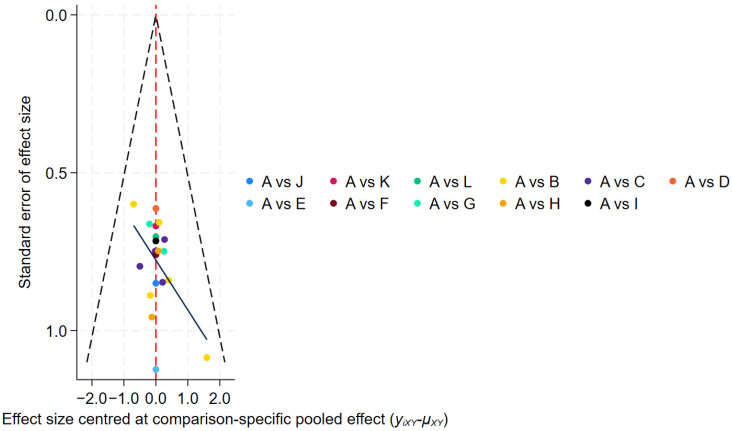
Efficiency Funnel Chart.

Publication bias assessment (Figure 7) showed a symmetrical funnel plot, indicating no significant small-study effects.

### 3.5. Tremor Six Score

**Figure 8:**
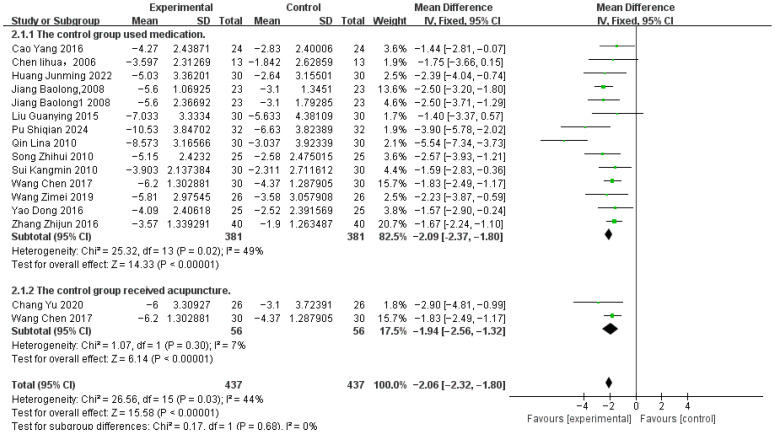
Tremor six score Correlation Chart [22,24,25,26,27,28,30,31,32,34,35,36,37,38,39,40].

**Figure 9:**
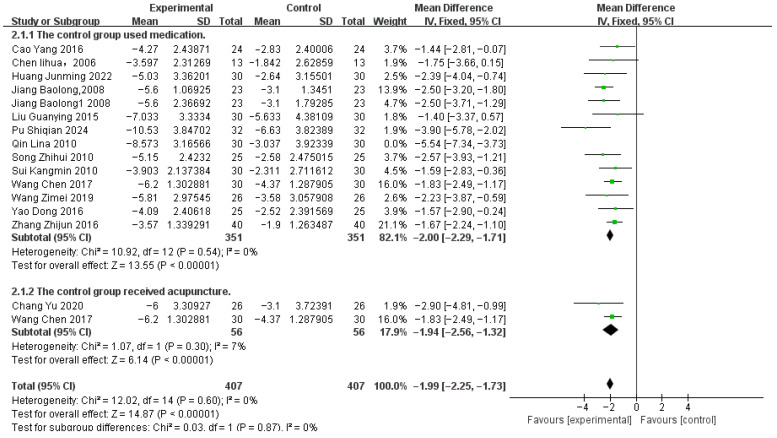
Research image after removal of associated study elements [22,24,25,26,28,30,31,32,34,35,36,37,38,39,40].

The Tremor Six Score was derived from a study by Louis ED et al. [42]. Data from 16 studies reporting this outcome measure were pooled, involving 407 patients in the acupuncture-related treatment groups and 407 patients in the control groups (Figure 8). Subgroup analyses were performed based on differences in control group types. Moderate heterogeneity was observed (*p* = 0.03, I^2^ = 44%), and a fixed-effects model was used. Sensitivity analysis identified study [27] as the source of heterogeneity; results after exclusion are shown in Figure 9.

The meta-analysis showed that, compared with control, acupuncture-related therapies significantly improved Tremor Six scores [MD −1.99, 95% CI (−2.25, −1.73), *p* < 0.00001].

Per-treatment analysis (Table 3) showed that, compared with control, four treatment modalities significantly improved outcomes:

Acupuncture (A) [MD = −3.08, 95% CI (−4.44, −1.73)]

Acupuncture + Propranolol (A+P) [MD = −1.68, 95% CI (−2.75, −0.60)]

Acupoint Catgut Embedding (ACE) [MD = −2.57, 95% CI (−4.78, −0.36)]

Acupuncture + Electroacupuncture + Propranolol (A+E+P) [MD = −2.13, 95% CI (−3.67, −0.59)]

Three modalities showed no statistically significant difference compared to control:

Acupuncture + Scalp Acupuncture + Propranolol (A+S+P) [MD = −1.57, 95% CI (−3.25, 0.11)]

Acupoint Catgut Embedding + Electroacupuncture (ACE+E) [MD = −2.23, 95% CI (−4.62, 0.16)]

Acupuncture + Tianma Gouteng Drink + Propranolol (A+TGD+P) [MD = −1.67, 95% CI (−3.50, 0.16)]

**Figure 10:**
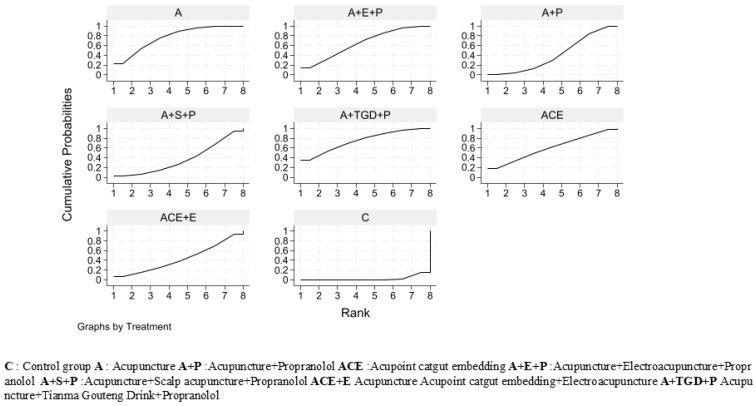
Tremor six score assessment scale (SUCRA) chart.

Based on SUCRA values, Acupuncture (A, 76.6%) and Acupuncture + Tianma Gouteng Drink + Propranolol (A+TGD+P, 75.1%) ranked highest, while Control (C, 2.2%) ranked lowest. The full ranking list is presented in Figure 10.

**Figure 11:**
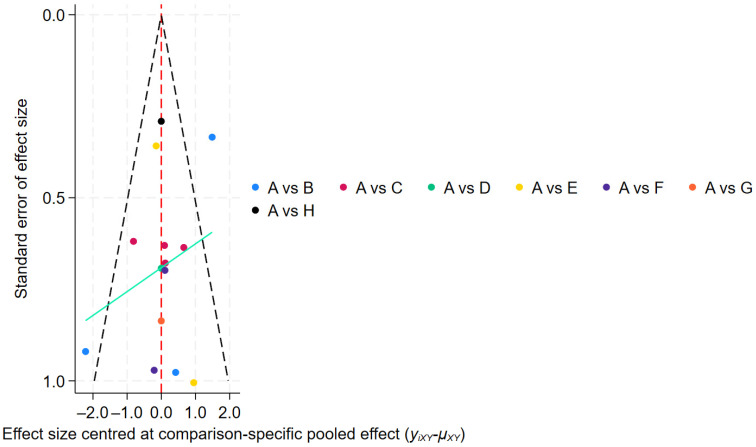
Tremor six score Funnel Chart.

Publication bias assessment (Figure 11) showed that most studies were evenly distributed on both sides of the midline; however, two studies fell outside the funnel plot boundaries, suggesting potential publication bias or small-study effects.

### 3.6. Adverse Events

**Figure 12:**
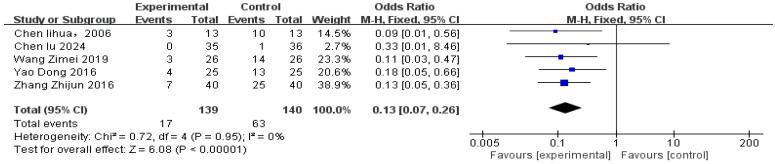
Comparison Chart of Adverse Events [22,23,24,25,26].

Based on five studies that reported comparisons of safety outcomes between the treatment group and the control group, the incidence of adverse events was analyzed (Figure 12). The safety analysis included a total of 279 patients, comprising 139 in the treatment group and 140 in the control group.

There were differences in the adverse events reported across the studies:

Chen Lihua et al. [22] and Zhang Zhijun et al. [26] reported gastrointestinal reactions (such as nausea and abdominal discomfort), bradycardia, and decreased blood pressure.

Chen Lu [23] reported subcutaneous hematoma.

Wang Zimei [24] and Yao Dong et al. [25] reported dizziness, bradycardia, and other unspecified adverse events.

No significant heterogeneity was detected (*p* = 0.95, I^2^ = 0%), so a fixed-effects model was appropriate. Due to the small number of included studies, no subgroup analysis was performed.

Overall, compared with the control group, the risk of adverse events was significantly reduced with acupuncture-related interventions, with a risk reduction of approximately 87% [RR 0.13, 95% CI (0.07, 0.25), *p* < 0.00001].

Analysis by treatment regimen (Table 2) showed that four treatment regimens had significantly better safety profiles than the control group:

Acupuncture + Propranolol (A+P) [OR = 0.18, 95% CI (0.05, 0.66)]

Acupuncture + Scalp Acupuncture + Propranolol (A+S+P) [OR = 0.09, 95% CI (0.01, 0.56)]

Acupoint Catgut Embedding + Electroacupuncture (ACE+E) [OR = 0.11, 95% CI (0.03, 0.47)]

Acupuncture + Tianma Gouteng Drink + Propranolol (A+TGD+P) [OR = 0.13, 95% CI (0.05, 0.36)]

One treatment combination did not show a statistically significant difference: Electroacupuncture + Auricular Acupuncture (E+AA) [OR = 0.33, 95% CI (0.01, 8.46)].

**Figure 13:**
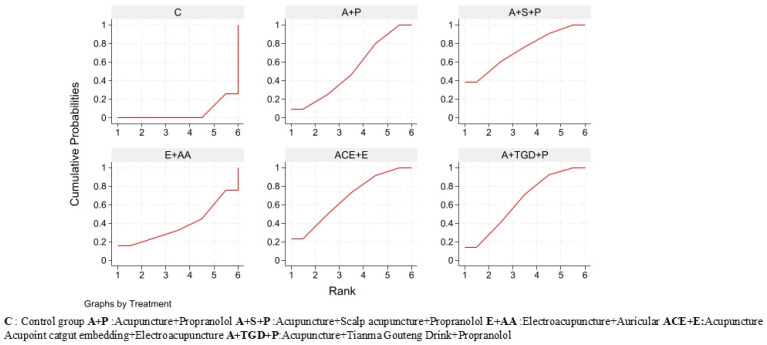
Adverse Events SUCRA Chart.

Based on SUCRA values, Acupuncture + Scalp Acupuncture + Propranolol (A+S+P, 73.1%) had the highest probability of being the safest intervention, while Control (C, 5.3%) ranked lowest. SUCRA values for the remaining interventions ranged from 38.4% to 67.8% (see Figure 13).

**Figure 14:**
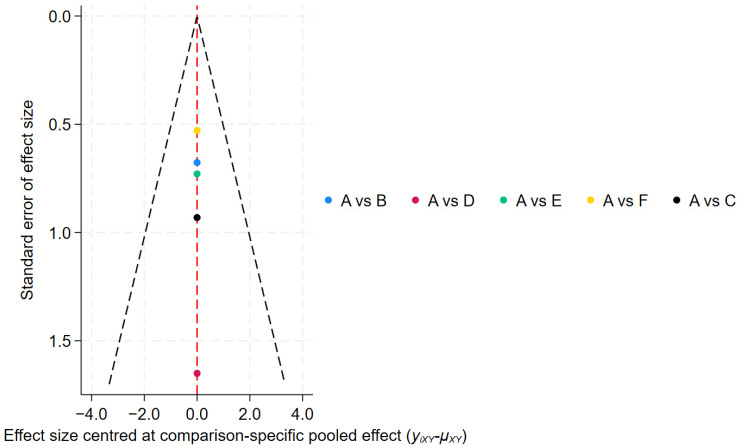
Adverse Events Funnel Chart.

Publication bias was assessed using a funnel plot (Figure 14); however, due to the limited number of studies (fewer than 10), this analysis may lack sufficient statistical power to detect asymmetry, and therefore the results should be interpreted with caution.

### 3.7. Recurrence Rate

The superior long-term efficacy of the treatment group was sustained at the one-month follow-up, as reported by Chen Lu et al. [23] (*p* < 0.001). Huang Junming et al. [35] reported follow-up results at three months, indicating that the treatment group had an efficacy rate of 6.7% after three months of treatment, while the control group had an efficacy rate of 0% after three months of treatment. When compared at the three-month follow-up, the two groups showed no significant difference in response rates (*p* > 0.05). However, Wang Chen et al. [30] reported a recurrence rate of 13.8% in the treatment group and 45.5% in the control group after three months of follow-up. The treatment group demonstrated a significantly lower recurrence rate compared to the control group (*p* < 0.05).

## 4. Discussion

### 4.1. Key Findings

This network meta-analysis synthesized data from 20 randomized controlled trials (RCTs) involving 1067 patients to comprehensively evaluate various acupuncture-related interventions for essential tremor (ET). The pooled results indicate that, compared with control, acupuncture-related therapies significantly improved the response rate [RR 4.36, 95% CI (3.14, 6.03), *p* < 0.00001], with consistent effects across studies (I^2^ = 0%). Regarding the Tremor Six score, despite moderate heterogeneity (I^2^ = 44%), acupuncture-related therapies also demonstrated significant improvement [MD −1.99, 95% CI (−2.25, −1.73), *p* < 0.00001]. Regarding safety, compared with control, acupuncture-related interventions significantly reduced the risk of adverse events [RR 0.13, 95% CI (0.07, 0.25), *p* < 0.00001], with no heterogeneity detected (I^2^ = 0%).

Per-treatment analysis showed that, compared with control, nine treatment modalities significantly improved efficacy, with scalp acupuncture (S) ranking highest in SUCRA value (81.5%). Regarding the Tremor Six score, acupuncture (A) ranked highest (76.6%). Regarding safety, acupuncture + scalp acupuncture + propranolol (A+S+P) had the highest probability of being the safest intervention (SUCRA 73.1%). These interventions encompassed various techniques, including acupuncture, electroacupuncture, thread embedding, and auricular acupuncture. Key findings included: (a) Nine treatment modalities significantly improved overall efficacy, with scalp acupuncture (S) showing the most pronounced effect. (b) Among the four therapies, acupuncture (A) was most effective in improving the Tremor Six score. (c) The acupuncture + scalp acupuncture + propranolol (A+S+P) A+S+P combination therapy reported the fewest adverse events.

### 4.2. Interpretation of Findings

The pathogenesis of ET remains incompletely understood, with three primary hypotheses proposed: (a) the neurodegenerative hypothesis, (b) the central oscillatory network hypothesis, and (c) the GABAergic hypothesis [4,5]. In experimental studies, Therapy S was shown to enhance the activity of the neurotransmitter gamma-aminobutyric acid (GABA) and its receptors (GABAA and GABAB) [14,15]. GABA neurotransmitter deficiency is considered closely associated with ET [6], and GABA A receptor α1 knockout mice exhibit the typical postural and action tremors characteristic of ET [43]. Regarding A therapy, it elevates GABA levels [44], aligning with Hypothesis c. Concurrently, studies indicate that both ET and Parkinson’s disease correlate with heightened cerebellar-thalamic-cortical circuit activity [5], and A therapy modulates this circuit activity [13], supporting Hypothesis b. Proponents of Hypothesis A emphasize that ET primarily affects middle-aged and elderly individuals, progresses slowly, and correlates with Parkinson’s disease and dementia incidence [5]. A treatment targeting neurodegenerative changes aligns with Hypothesis A [45]. Regarding adverse reactions, fewer adverse events are reported with the Acupuncture + Propranolol (A+P) combination. Common adverse effects of the first-line drug propranolol, such as bradycardia and bronchospasm [11], are also relatively modulated by acupuncture [46,47,48], suggesting this may be a potential mechanism for acupuncture’s reduction in adverse reactions.

### 4.3. Future Research Directions

The optimal acupuncture points for treatment remain unstandardized, and the mechanisms underlying acupuncture therapy for this disease are not fully understood. Future research should focus on elucidating specific therapeutic mechanisms. There is a need for more rigorously conducted RCTs with systematic follow-up to directly compare recurrence rates between interventions.

### 4.4. Advantages and Limitations

This network meta-analysis has several strengths, including its comprehensive evaluation of multiple acupuncture-related interventions for tremor disorders and the use of rigorous methodology to compare both efficacy and safety outcomes. However, several limitations should be considered when interpreting our findings.

First, the methodological quality of the included randomized controlled trials (RCTs) raises concerns regarding risk of bias. A total of 20 RCTs were included in this analysis, with sample sizes ranging from 26 to 80 patients per trial, indicating that all studies were relatively small. Due to the inherent nature of acupuncture interventions—particularly those involving needle insertion or electrical stimulation—blinding of practitioners was not feasible, and blinding of participants was challenging, introducing high risk of performance bias. Furthermore, none of the included studies reported adequate allocation concealment, and many failed to clearly describe the methods used for random sequence generation. Consequently, according to Cochrane risk of bias criteria, the majority of domains across the included studies were rated as having high or unclear risk of bias. This methodological limitations may have overestimated the true treatment effects and should be considered when applying our findings to clinical practice.

Second, data sparsity for certain intervention nodes limits the precision and stability of our estimates. Interventions such as Electroacupuncture + Auricular (E+AA) and acupoint catgut embedding (ACE) were supported by a limited number of studies, resulting in wide confidence intervals and uncertain treatment rankings. Similarly, the small number of studies reporting recurrence rates and adverse events precluded robust subgroup analyses and limited our ability to draw definitive conclusions regarding long-term outcomes and safety profiles. These gaps highlight the need for additional high-quality RCTs to strengthen the evidence base for these specific interventions.

Third, inconsistencies between direct and indirect comparisons may impact the robustness of the network meta-analysis results. Although we assessed consistency using standard methods, the limited number of studies for certain pairwise comparisons reduced the statistical power to detect true inconsistency. Therefore, findings derived primarily from indirect evidence should be interpreted with particular caution regarding their generalizability.

Fourth, all studies included in this analysis were conducted in China, which may limit the generalizability of our findings to other populations and healthcare settings. While this geographic concentration reflects the widespread clinical use of acupuncture-related therapies in China, cultural differences, variations in treatment protocols, and population-specific factors may influence treatment effects in other regions.

Finally, substantial heterogeneity was observed for some outcome measures, which could not be fully explained by subgroup analyses due to the limited number of available studies. This heterogeneity may reflect variations in treatment duration, acupuncture point selection, stimulation techniques, or patient characteristics across the included trials.

Despite these limitations—particularly the low-to-moderate overall level of evidence across the included studies—the superiority demonstrated by acupuncture-related interventions warrants consideration. The following factors may explain this finding: (1) the consistency of treatment effects across multiple studies, coupled with extremely low heterogeneity in key outcome measures (e.g., I^2^ = 0% for response rate), suggests that this positive signal is robust; (2) mechanistic studies support its biological plausibility, linking acupuncture to GABA modulation and regulation of the cerebellar-thalamic-cortical circuit [14,15,44]. Nevertheless, given the methodological shortcomings of the included studies, these results should be regarded as preliminary evidence and require validation through well-designed, large-scale, well-blinded, multicenter randomized controlled trials (RCTs) with long-term follow-up.

In summary, while this network meta-analysis provides valuable evidence supporting the efficacy and safety of acupuncture-related interventions for tremor disorders, the findings should be interpreted in light of the high risk of bias in the included RCTs, data sparsity for certain nodes, geographic limitations, and observed heterogeneity. Future large-scale, rigorously designed, and well-blinded multicenter RCTs with extended follow-up periods are urgently needed to validate our findings and enhance their generalizability to broader clinical populations.

## 5. Conclusions

This network meta-analysis indicates that acupuncture-related interventions are effective and safe for the treatment of essential tremor (ET). Based on SUCRA values, scalp acupuncture (S) was most effective in improving treatment response rates, while acupuncture (A) was most effective in reducing Tremor Six scores. Regarding safety, the acupuncture combined with propranolol (A+P) regimen was associated with the lowest risk of adverse events.

Overall, acupuncture-related therapies offer several advantages, including fewer complications, high safety, good accessibility, and the ability to significantly improve ET symptoms and treatment-related adverse events.

However, these results should be interpreted with caution due to methodological limitations in the included randomized controlled trials (small sample sizes, lack of blinding, inadequate allocation concealment), sparse data for some interventions, the geographic concentration of studies within China (which limits generalizability), and the low-to-moderate quality of the included evidence.

These findings provide evidence-based guidance for clinicians in selecting appropriate acupuncture interventions for ET management while also highlighting the need for future high-quality randomized controlled trials to validate these results.

## Figures and Tables

**Table 1:** General Overview of Related Research.

Study	Sample Size	Sex (Male/Female)		Year		Intervention		Duration Of Use	Outcome
	C/T	C	T	C	T	C	T		
**Chen lihua, 2006** [22]	13/13	NA	NA	61.5 ± 4.4	58.8 ± 5.1	Propranolol	Acupuncture + Scalp acupuncture + Propranolol	1.5 months	A, B, C
**Huang Junming 2022** [35]	30/30	16/14	15/15	65.83 ± 9.12	62.63 ± 7.84	Propranolol	Scalp Acupuncture + Propranolol	1 month	A, B, D, E
**Jiang Baolong 2008** [36]	23/23	11/12	15/8	51.11 ± 10.62	49.91 ± 11.85	Propranolol	Acupuncture + Electroacupuncture + Propranolol	1.5 months	A, B
**Jiang Baolong 2008** [37]	23/23	12/11	14/9	51.3	50.4	Propranolol	Acupuncture + Propranolol	1.5 months	A, B
**Liu Guanying 2015** [38]	30/30	9/21	14/16	54.03 ± 11.15	55.57 ± 13.07	Propranolol	Acupuncture + Electroacupuncture + Propranolol	3 months	A, B
**Sui Kangmin 2010** [40]	30/30	16/14	18/12	60.2 ± 6.3	59.7 ± 5.9	Propranolol	Acupuncture + Propranolol	1 month	A, B
**Wang Fei 2010** [31]	18/18	NA	NA	62.3	62.3	Propranolol	Acupuncture + Propranolol	2 months	B, F
**Yao Dong 2016** [25]	25/25	12/13	14/11	60 ± 7	58 ± 8	Propranolol	Acupuncture + Propranolol	1 month	A, B, C
**Zhang Yunjian 2022** [33]	20/26	11/9	14/12	65.6 ± 6.8	64.3 ± 7.8	Propranolol	Acupuncture + Massage (Tuina) + Siping Dingchan Drink +Propranolol	2 months	B, E
**Zhang Zhijun 2016** [26]	40/40	18/22	19/21	65. 10 ± 8.22	66.35 ± 8.19	Propranolol	Acupuncture + Tianma Gouteng Drink + Propranolol	2 months	A, B, C
**Cao Yang 2016** [32]	24/24	14/10	15/9	NA	NA	Propranolol	Acupuncture + Scalp acupuncture + Propranolol	1.5 months	A, B
**Song Zhihui 2010** [28]	25/25	13/12	14/11	52	51	Propranolol	Acupoint catgut embedding	3 months	A, B
**Pu Shiqian 2024** [39]	34/34	13/19	15/17	62.22 ± 5.65	62.50 ± 6.51	Propranolol	Acupuncture	3 months	A, B, E
**Qin Lina 2010** [27]	30/30	NA	NA	48.9	48.9	Propranolol	Acupuncture	1 month	A, B
**Sun Gangyuan 2016** [29]	20/20	12/8	11/9	56.24 ± 8.69	55.22 ± 9.34	Propranolol	Scalp Acupuncture	1 month	A, B
**Wang Zimei 2019** [24]	26/26	11/15	14/12	NA	NA	Propranolol	Acupoint catgut embedding + Electroacupuncture	3 months	A, B, C
**Chen lu 2024** [23]	39/39	16/20	17/18	54.94 ± 6.58	57.31 ± 10.45	Acupuncture	Electroacupuncture + Auricular Acupuncture	1 month	B, C, D, G, I
**Wang Song 2022** [41]	30/30	13/17	17/13	66.57 ± 10.43	69.67 ± 11.06	Acupuncture	Acupuncture	half a month	B, G, I, J
**Chang Yu 2020** [34]	26/26	16/10	14/12	50.15 ± 11.03	51.12 ± 11.24	Acupuncture	Acupuncture	1.5 months	A, B, C, D, K,
**Wang Chen 2017** [30]	30/30	17/13	14/16	60 ± 9	58 ± 8	Acupuncture	Acupuncture	1 month	A, B, H

**Note**: C: Control group, T: Treatment group, NA: Not Available. **Outcome Key**: A: Tremor Six Score, B: Efficacy, C: Adverse Reactions, D: Hamilton Depression Scale, E: Tremor Grading, F: Fahn-Tolosa-Marin Tremor Rating Scale, G: Quality of Life Scale, H: Depression Rating, I: The Tremor Research Group Essential Tremor Rating Assessment Scale, J: Bain-Findley Tremor Rating Scale, K: Traditional Chinese Medicine Syndrome Score.

**Table 2:** Efficacy and Adverse Reaction League Table.

**A**	-	-	-	-	-	-	-	-	-	-	-
0.65 (0.24, 1.78)	**A+P**	-	-	-	-	0.51 (0.05, 4.88)	1.90 (0.06, 62.51)	0.64 (0.09, 4.47)	-	0.72 (0.13, 3.90)	0.18 (0.05, 0.66)
0.89 (0.23, 3.52)	1.37 (0.33, 5.67)	**S+P**	-	-	-	-	-	-	-	-	-
0.27 (0.03, 2.68)	0.41 (0.04, 4.24)	0.30 (0.02, 3.70)	**S**	-	-	-	-	-	-	-	-
1.19 (0.23, 6.10)	1.84 (0.35, 9.77)	1.34 (0.20, 9.08)	4.44 (0.31, 63.33)	**ACE**	-	-	-	-	-	-	-
0.86 (0.26, 2.79)	1.32 (0.39, 4.52)	0.96 (0.21, 4.52)	3.19 (0.29, 35.38)	0.72 (0.12, 4.25)	**A+E+P**	-	-	-	-	-	-
0.87 (0.23, 3.32)	1.35 (0.34, 5.35)	0.98 (0.19, 5.20)	3.25 (0.27, 39.06)	0.73 (0.11, 4.82)	1.02 (0.23, 4.61)	**A+S+P**	0.27 (0.01, 11.07)	0.81 (0.08, 8.17)	-	0.71 (0.09, 5.77)	0.09 (0.01, 0.56)
0.96 (0.20, 4.53)	1.48 (0.30, 7.26)	1.08 (0.17, 6.82)	3.56 (0.26, 48.46)	0.80 (0.10, 6.21)	1.12 (0.20, 6.16)	1.09 (0.18, 6.74)	**E+AA**	2.98 (0.09, 102.34)	-	2.62 (0.09, 78.19)	0.33 (0.01, 8.46)
0.64 (0.11, 3.84)	0.98 (0.16, 6.12)	0.72 (0.09, 5.59)	2.38 (0.15, 37.53)	0.53 (0.06, 4.99)	0.74 (0.11, 5.12)	0.73 (0.10, 5.54)	0.67 (0.08, 5.89)	**ACE+E**	-	0.88 (0.15, 5.13)	0.11 (0.03, 0.47)
0.50 (0.12, 2.19)	0.78 (0.17, 3.53)	0.57 (0.10, 3.35)	1.88 (0.14, 24.31)	0.42 (0.06, 3.07)	0.59 (0.12, 3.01)	0.58 (0.10, 3.31)	0.53 (0.08, 3.59)	0.79 (0.09, 6.58)	**A+M+SDD+P**	-	-
0.83 (0.18, 3.82)	1.28 (0.27, 6.14)	0.93 (0.15, 5.78)	3.08 (0.23, 41.33)	0.69 (0.09, 5.27)	0.97 (0.18, 5.21)	0.95 (0.16, 5.71)	0.86 (0.12, 6.18)	1.30 (0.15, 11.26)	1.64 (0.25, 10.99)	**A+TGD+P**	0.13 (0.05, 0.36)
3.40 (1.75, 6.63)	5.25 (2.46, 11.16)	3.82 (1.15, 12.71)	12.67 (1.40, 114.42)	2.85 (0.64, 12.64)	3.97 (1.50, 10.50)	3.89 (1.23, 12.35)	3.56 (0.87, 14.47)	5.33 (1.01, 28.21)	6.75 (1.82, 25.03)	4.11 (1.04, 16.29)	**C**

Note: This table presents a league table that simultaneously displays comparisons for both efficacy outcomes and adverse events. Values represent odds ratios (OR) with 95% confidence intervals (CI) shown in parentheses. Lower-left section (below the diagonal): Presents comparisons for efficacy outcomes. OR > 1 favors the row intervention (indicating higher response rate or better efficacy). Upper-right section (above the diagonal): Presents comparisons for adverse events. OR < 1 favors the row intervention (indicating lower risk of adverse events). A = Acupuncture, A+P = Acupuncture + Propranolol, **S+P** = Scalp Acupuncture + Propranolol, **S** = Scalp Acupuncture, ACE = Acupoint Implantation Therapy, A+E+P = Acupuncture + Electroacupuncture + Propranolol, A+S+P = Acupuncture + Scalp Acupuncture + Propranolol, ACE+E = Acupoint Implantation Therapy + Electroacupuncture, A+TGD+P = Acupuncture + Gastrodia and Polygonum Decoction + Propranolol. C = Control group.

**Table 3:** League Table for Tremor Six Score.

**A**	1.41 (−0.32, 3.13)	0.51 (−2.07, 3.10)	0.96 (−1.13, 3.04)	1.51 (−0.64, 3.66)	0.85 (−1.89, 3.60)	1.41 (−0.86, 3.69)	3.08 (1.73, 4.44)
−1.41 (−3.13, 0.32)	**A+P**	−0.89 (−3.35, 1.56)	−0.45 (−2.32, 1.42)	0.10 (−1.89, 2.10)	−0.55 (−3.17, 2.07)	0.01 (−2.11, 2.13)	1.68 (0.60, 2.75)
−0.51 (−3.10, 2.07)	0.89 (−1.56, 3.35)	**ACE**	0.44 (−2.25, 3.13)	1.00 (−1.78, 3.77)	0.34 (−2.91, 3.59)	0.90 (−1.97, 3.77)	2.57 (0.36, 4.78)
−0.96 (−3.04, 1.13)	0.45 (−1.42, 2.32)	−0.44 (−3.13, 2.25)	**A+E+P**	0.56 (−1.72, 2.83)	−0.10 (−2.94, 2.74)	0.46 (−1.93, 2.85)	2.13 (0.59, 3.67)
−1.51 (−3.66, 0.64)	−0.10 (−2.10, 1.89)	−1.00 (−3.77, 1.78)	−0.56 (−2.83, 1.72)	**A+S+P**	−0.66 (−3.58, 2.27)	−0.10 (−2.58, 2.39)	1.57 (−0.11, 3.25)
−0.85 (−3.60, 1.89)	0.55 (−2.07, 3.17)	−0.34 (−3.59, 2.91)	0.10 (−2.74, 2.94)	0.66 (−2.27, 3.58)	**ACE+E**	0.56 (−2.45, 3.57)	2.23 (−0.16, 4.62)
−1.41 (−3.69, 0.86)	−0.01 (−2.13, 2.11)	−0.90 (−3.77, 1.97)	−0.46 (−2.85, 1.93)	0.10 (−2.39, 2.58)	−0.56 (−3.57, 2.45)	**A+TGD+P**	1.67 (−0.16, 3.50)
−3.08 (−4.44, −1.73)	−1.68 (−2.75, −0.60)	−2.57 (−4.78, −0.36)	−2.13 (−3.67, −0.59)	−1.57 (−3.25, 0.11)	−2.23 (−4.62, 0.16)	−1.67 (−3.50, 0.16)	**C**

Note: League table for Tremor Six Score. Values are mean difference (MD) with 95% CI. MD < 0 favors row intervention (greater tremor reduction); MD > 0 favors column intervention. Bold indicates statistical significance (95% CI excludes 0). Lower-left and upper-right sections are symmetrical (negatives of each other). A = Acupuncture, A+P = Acupuncture + Propranolol, ACE = Acupoint Implantation Therapy, A+E+P = Acupuncture + Electroacupuncture + Propranolol, A+S+P = Acupuncture + Scalp Acupuncture + Propranolol, ACE+E = Acupoint Implantation Therapy + Electroacupuncture, A+TGD+P = Acupuncture + Gastrodia and Polygonum Decoction + Propranolol. C = Control group.

## Data Availability

No new data were created or analyzed in this study. Data sharing is not applicable to this article.

## Supplementary Materials

The following supporting information can be downloaded at: https://www.mdpi.com/article/10.3390/healthcare14060803/s1, Supplementary Materials File S1: Search Query and Results

## Abbreviations

**ET** = Essential tremor, **CNKI** = China National Knowledge Infrastructure, **CBM** = China Biomedical Database, **C** =Control group, **A** = Acupuncture, **A+P** =Acupuncture + Propranolol, **S+P** = Scalp Acupuncture + Propranolol, **S** = Scalp Acupuncture, **ACE** =Acupoint catgut embedding, **A+E+P** =Acupuncture + Electroacupuncture + Propranolol, **A+S+P** =Acupuncture + Scalp acupuncture + Propranolol, **E+AA** =Electroacupuncture + Auricular, **ACE+E** = Acupuncture Acupoint catgut embedding + Electroacupuncture, **A+M+SDD+P** = Acupuncture + Massage(Tuina) + Siping Dingchan Drink +Propranolol, **A+TGD+P** = Acupuncture + Tianma Gouteng Drink + Propranolol. **SUCRA** = Surface under the Cumulative Ranking Curve, **FDA** = Drug Administration, **DBS** = deep brain stimulation, **MRIgFUS** = MRI-guided high-intensity focused ultrasound, **NMA** = network meta-analysis, **RCTs** = Randomized controlled trials, **GABA** = gamma-aminobutyric acid.
