# Supplementary material for: Comparative Efficacy of Acupuncture Therapy in Primary Essential Tremor: A Network Meta-Analysis and Systematic Review
Source: Healthcare (Basel). 2026 Mar 21;14(6):803. doi: 10.3390/healthcare14060803 (PMC13026131; doi:10.3390/healthcare14060803)
Supplement: Supplementary file 1 [file healthcare-14-00803-s001.zip › healthcare-4175128-supplementary.pdf]

Embase

Session Results

....

No.	Query Results	Results	Date
#24.	('essential tremor'/exp OR ('essential tremors' OR 'tremor, essential' OR 'tremors, essential' OR 'benign essential tremor' OR 'benign essential tremors' OR 'essential tremor, benign' OR 'essential tremors, benign' OR 'tremor, benign essential' OR 'tremors, benign essential' OR 'familial tremors' OR 'tremor, familial' OR 'tremors, familial' OR 'hereditary essential tremor' OR 'familial tremor' OR 'hereditary tremor')) AND (('acupuncture'/exp OR 'acupuncture point'/exp) OR ('acupuncture'/exp OR 'acupuncture point'/exp OR 'points, acupuncture') OR ('acupuncture treatment' OR 'acupuncture treatments' OR 'treatment, acupuncture' OR 'therapy, acupuncture' OR 'pharmacoacupuncture treatment' OR 'treatment, pharmacoacupuncture' OR 'pharmacoacupuncture therapy' OR 'therapy, pharmacoacupuncture' OR 'acupotomy' OR 'acupotomies') OR 'electroacupuncture'/exp OR 'body meridian'/exp OR acupuncture:ab,ti OR 'electroacupuncture':ab,ti OR acupoint:ab,ti OR meridian:ab,ti OR deqi:ab,ti) AND ('clinical trial'/exp OR random*:ab OR 'controlled clinical	3	19 Oct 2025

trial'/exp OR 'controlled clinical trial':ab,ti OR 'randomized controlled trial'/exp OR 'randomized controlled trial':ab,ti OR placebo:ab OR trial:ti)		
#23. 'clinical trial'/exp OR random*:ab OR 'controlled clinical trial'/exp OR 'controlled clinical trial':ab,ti OR 'randomized controlled trial'/exp OR 'randomized controlled trial':ab,ti OR placebo:ab OR trial:ti	4,143,050	19 Oct 2025
#22. trial:ti	574,746	19 Oct 2025
#21. placebo:ab	447,102	19 Oct 2025
#20. 'randomized controlled trial':ab,ti	207,897	19 Oct 2025
#19. 'randomized controlled trial'/exp	1,119,388	19 Oct 2025
#18. 'controlled clinical trial':ab,ti	37,337	19 Oct 2025
#17. 'controlled clinical trial'/exp	1,310,028	19 Oct 2025
#16. random*:ab	2,410,515	19 Oct 2025
#15. 'clinical trial'/exp	2,632,915	19 Oct 2025
#14. ('acupuncture'/exp OR 'acupuncture point'/exp) OR ('acupuncture'/exp OR 'acupuncture point'/exp OR 'points, acupuncture') OR ('acupuncture treatment' OR 'acupuncture treatments' OR 'treatment, acupuncture' OR 'therapy, acupuncture' OR 'pharmacoacupuncture treatment' OR 'treatment, pharmacoacupuncture' OR 'pharmacoacupuncture therapy' OR 'therapy,	78,667	19 Oct 2025

	pharmacoacupuncture' OR 'acupotomy' OR 'acupotomies') OR 'electroacupuncture'/exp OR 'body meridian'/exp OR acupuncture:ab,ti OR 'electroacupuncture':ab,ti OR acupoint:ab,ti OR meridian:ab,ti OR deqi:ab,ti		
#13.	deqi:ab,ti	366	19 Oct 2025
#12.	meridian:ab,ti	7,221	19 Oct 2025
#11.	acupoint:ab,ti	6,193	19 Oct 2025
#10.	'electroacupuncture':ab,ti	10,218	19 Oct 2025
#9.	acupuncture:ab,ti	45,874	19 Oct 2025
#8.	'body meridian'/exp	2,061	19 Oct 2025
#7.	'electroacupuncture'/exp	11,845	19 Oct 2025
#6.	'acupuncture treatment' OR 'acupuncture treatments' OR 'treatment, acupuncture' OR 'therapy, acupuncture' OR 'pharmacoacupuncture treatment' OR 'treatment, pharmacoacupuncture' OR 'pharmacoacupuncture therapy' OR 'therapy, pharmacoacupuncture' OR 'acupotomy' OR 'acupotomies'	7,410	19 Oct 2025
#5.	'acupuncture'/exp OR 'acupuncture point'/exp OR 'points, acupuncture'	70,127	19 Oct 2025
#4.	'acupuncture'/exp OR 'acupuncture point'/exp	70,125	19 Oct 2025

#3.	'essential tremor'/exp OR ('essential tremors' OR 'tremor, essential' OR 'tremors, essential' OR 'benign essential tremor' OR 'benign essential tremors' OR 'essential tremor, benign' OR 'essential tremors, benign' OR 'tremor, benign essential' OR 'tremors, benign essential' OR 'familial tremors' OR 'tremor, familial' OR 'tremors, familial' OR 'hereditary essential tremor' OR 'familial tremor' OR 'hereditary tremor')	9,442	19 Oct 2025
#2.	'essential tremors' OR 'tremor, essential' OR 'tremors, essential' OR 'benign essential tremor' OR 'benign essential tremors' OR 'essential tremor, benign' OR 'essential tremors, benign' OR 'tremor, benign essential' OR 'tremors, benign essential' OR 'familial tremors' OR 'tremor, familial' OR 'tremors, familial' OR 'hereditary essential tremor' OR 'familial tremor' OR 'hereditary tremor'	458	19 Oct 2025
#1.	'essential tremor'/exp	9,304	19 Oct 2025

Search Name:

Date Run: 19/10/2025 04:06:02

Comment:

the Cochrane Library

#1 MeSH descriptor: [Acupuncture] explode all trees 216

#2 MeSH descriptor: [Acupuncture Therapy] explode all trees 7295

#3 MeSH descriptor: [Electroacupuncture] explode all trees 1207

#4 MeSH descriptor: [Acupuncture Points] explode all trees 2904

#5 (Acupuncture):ti,ab,kw (Word variations have been searched) 22195

#6 (Acupoint*):ti,ab,kw (Word variations have been searched) 6753

#7 (Meridian*):ti,ab,kw (Word variations have been searched) 1720

#8 (Electroacupuncture):ti,ab,kw (Word variations have been searched) 4117

#9 (Electro-acupuncture):ti,ab,kw (Word variations have been searched) 739

#10 MeSH descriptor: [Needles] explode all trees 1636

#11 (Acupunctur):ti,ab,kw (Word variations have been searched) 22189

#12 (Needling):ti,ab,kw (Word variations have been searched) 25630

#13 MeSH descriptor: [Essential Tremor] explode all trees 205

#14 (Essential Tremors):ti,ab,kw (Word variations have been searched) 593

#15 (Tremor, Essential):ti,ab,kw (Word variations have been searched) 593

#16 (Tremors, Essential):ti,ab,kw (Word variations have been searched) 593

#17 (Benign Essential Tremor):ti,ab,kw (Word variations have been searched) 12

#18 (Benign Essential Tremors):ti,ab,kw (Word variations have been searched) 12

#19 (Essential Tremor, Benign):ti,ab,kw (Word variations have been searched) 12

#20 (Essential Tremors, Benign):ti,ab,kw (Word variations have been searched) 12

#21 (Tremor, Benign Essential):ti,ab,kw (Word variations have been searched) 12

#22 (Tremors, Benign Essential):ti,ab,kw (Word variations have been searched) 12

#23 (Familial Tremor):ti,ab,kw (Word variations have been searched) 67

#24 (Familial Tremors):ti,ab,kw (Word variations have been searched) 67

#25 (Tremor, Familial):ti,ab,kw (Word variations have been searched) 67

#26 (Tremors, Familial):ti,ab,kw (Word variations have been searched) 67

#27 (Hereditary Essential Tremor):ti,ab,kw (Word variations have been searched) 1

#28 #13 or #14 or #15 or #16 or #17 or #18 or #19 or #20 or #21 or #22 or #23 or

#24 or #25 or #26 or #27 652

#29 #1 or #2 or #3 or #4 or #5 or #6 or #7 or #8 or #9 or #10 or #11 0r #12 28104

#30 #28 and #29 4

PUBMED

#1"Essential Tremor"[Mesh]

#2((((((((((((Essential Tremors) OR (Tremor, Essential)) OR (Tremors, Essential)) OR (Benign Essential Tremor)) OR (Benign Essential Tremors)) OR (Essential Tremor, Benign)) OR (Essential Tremors, Benign)) OR (Tremor, Benign Essential)) OR (Tremors, Benign Essential)) OR (Familial Tremor)) OR (Familial Tremors)) OR (Tremor, Familial)) OR (Tremors, Familial)) OR (Hereditary Essential Tremor)

#3("Essential Tremor"[Mesh]) OR (((((((((((((Essential Tremors) OR (Tremor, Essential)) OR (Tremors, Essential)) OR (Benign Essential Tremor)) OR (Benign Essential Tremors)) OR (Essential Tremor, Benign)) OR (Essential Tremors, Benign)) OR (Tremor, Benign Essential)) OR (Tremors, Benign Essential)) OR (Familial Tremor)) OR (Familial Tremors)) OR (Tremor, Familial)) OR (Tremors, Familial)) OR (Hereditary Essential Tremor))

#4 #2 ((((("Acupuncture"[Mesh])) OR (Pharmacopuncture[Title/Abstract])) OR (((((((((("Acupuncture Therapy"[Mesh]) OR (Acupuncture Treatment[Title/Abstract])) OR (Acupuncture Treatments[Title/Abstract])) OR (Treatment, Acupuncture[Title/Abstract])) OR (Therapy, Acupuncture[Title/Abstract])) OR (Pharmacoacupuncture Treatment[Title/Abstract])) OR (Treatment, Pharmacoacupuncture[Title/Abstract])) OR (Pharmacoacupuncture Therapy[Title/Abstract])) OR (Therapy, Pharmacoacupuncture[Title/Abstract])) OR (Acupotomy[Title/Abstract])) OR (Acupotomies[Title/Abstract]))) OR (((((("Needles"[Mesh]) OR (Needle[Title/Abstract])) OR (Hypodermic Needles[Title/Abstract])) OR (Hypodermic Needle[Title/Abstract])) OR (Needle, Hypodermic[Title/Abstract])) OR (Needles, Hypodermic[Title/Abstract]))) OR (("Electroacupuncture"[Mesh]) OR (electro-acupuncture[Title/Abstract]))

#5 (#2 ((((("Acupuncture"[Mesh])) OR (Pharmacopuncture[Title/Abstract])) OR (((((((((("Acupuncture Therapy"[Mesh]) OR (Acupuncture Treatment[Title/Abstract])) OR (Acupuncture Treatments[Title/Abstract])) OR (Treatment, Acupuncture[Title/Abstract])) OR (Therapy, Acupuncture[Title/Abstract])) OR (Pharmacoacupuncture Treatment[Title/Abstract])) OR (Treatment, Pharmacoacupuncture[Title/Abstract])) OR (Pharmacoacupuncture Therapy[Title/Abstract])) OR (Therapy, Pharmacoacupuncture[Title/Abstract])) OR (Acupotomy[Title/Abstract])) OR

(Acupotomies[Title/Abstract]))) OR ((((("Needles"[Mesh]) OR (Needle[Title/Abstract])) OR (Hypodermic Needles[Title/Abstract])) OR (Hypodermic Needle[Title/Abstract])) OR (Needle, Hypodermic[Title/Abstract])) OR (Needles, Hypodermic[Title/Abstract]))) OR (("Electroacupuncture"[Mesh]) OR (electro-acupuncture[Title/Abstract]))) AND (("Essential Tremor"[Mesh]) OR ((((((((((((((Essential Tremors) OR (Tremor, Essential)) OR (Tremors, Essential)) OR (Benign Essential Tremor)) OR (Benign Essential Tremors)) OR (Essential Tremor, Benign)) OR (Essential Tremors, Benign)) OR (Tremor, Benign Essential)) OR (Tremors, Benign Essential)) OR (Familial Tremor)) OR (Familial Tremors)) OR (Tremor, Familial)) OR (Tremors, Familial)) OR (Hereditary Essential Tremor)))

Web of Science

1 ″TS=(essential tremor OR Essential Tremors OR Tremor, Essential OR
Tremors, Essential OR Benign Essential Tremor OR Benign Essential Tremors OR Essential Tremor, Benign OR Essential Tremors, Benign OR Tremor, Benign Essential OR Tremors, Benign Essential OR Familial Tremor OR Familial Tremors OR Tremor, Familial OR Tremors, Familial OR Hereditary Essential Tremor) ″

2 ″TS=(Acupuncture OR Electroacupuncture OR Pharmacopuncture) ″

3 ″TS=(Acupuncture Therapy OR Acupuncture Treatment OR Acupuncture Treatments OR Treatment, Acupuncture OR Therapy, Acupuncture OR Pharmacoacupuncture Treatment OR Treatment, Pharmacoacupuncture OR Pharmacoacupuncture Therapy OR Therapy, Pharmacoacupuncture) ″

4 ″TS=(Acupuncture Points OR Acupuncture Point OR Point, Acupuncture OR
Points, Acupuncture OR Acupoints OR Acupoint) ″

5 ″#4 OR #3 OR #2 ″

6 ″#5 AND #1 ″

VIP

(M=(特发性震颤 OR 原发性震颤 OR 家族性特发性震颤 OR 良性特发性震颤) AND M=(针刺 OR 电针 OR 耳针 OR 耳穴 OR 火针 OR 手针 OR 体针 OR 毫针 OR 温针 OR 头针 OR 腹针 OR 腕踝针 OR 眼针 OR 水针 OR 芒针 OR 埋针 OR 梅花针))

CNKI

TKA=(特发性震颤 OR 原发性震颤 OR 家族性特发性震颤 OR 良性特发性震颤 OR

颤证 OR ET) AND TKA=(针刺 OR 电针 OR 耳针 OR 耳穴 OR 火针 OR 手针 OR 体针 OR 毫针 OR 温针 OR 头针 OR 腹针 OR 腕踝针 OR 眼针 OR 水针 OR 芒针 OR 埋针 OR 埋线 OR 梅花针) AND FT=(临床)

Wanfang

主题:(特发性震颤 OR 原发性震颤 OR 家族性特发性震颤 OR 良性特发性震颤) and 主题:(针刺 OR 电针 OR 耳针 OR 耳穴 OR 火针 OR 手针 OR 体针 OR 毫针 OR 温针 OR 头针 OR 腹针 OR 腕踝针 OR 眼针 OR 水针 OR 芒针 OR 埋针 OR 埋线 OR 梅花针)

CBM

2) 临床 OR 随机对照 OR RCT OR 随机对照试验 6647216 2025-11-02 17:22:59.0

3) 针刺 OR 电针 OR 耳针 OR 耳穴 OR 火针 OR 手针 OR 体针 OR 毫针 OR 温针 OR 头针 OR 腹针 OR 腕踝针 OR 眼针 OR 水针 OR 芒针 OR 埋针 OR 梅花针 OR 埋线 271480 2025-11-02 17:23:24.0

5) 特发性震颤 OR 原发性震颤 OR 家族性特发性震颤 OR 良性特发性震颤 6204 2025-11-02 17:25:34.0

6) ((特发性震颤 OR 原发性震颤 OR 家族性特发性震颤 OR 良性特发性震颤) AND (针刺 OR 电针 OR 耳针 OR 耳穴 OR 火针 OR 手针 OR 体针 OR 毫针 OR 温针 OR 头针 OR 腹针 OR 腕踝针 OR 眼针 OR 水针 OR 芒针 OR 埋针 OR 梅花针 OR 埋线) AND (临床 OR 随机对照 OR RCT OR 随机对照试验)) AND %[常用字段] 40 2025-11-02 17:26:12.0
